# ASIC currents in cultured primate retinal amacrine/ganglion cells

**DOI:** 10.14814/phy2.70290

**Published:** 2025-05-12

**Authors:** Talib Saafir, Tiandong Leng, Koichi Inoue, Zhi‐Gang Xiong, Peter MacLeish

**Affiliations:** ^1^ Department of Neurobiology Neuroscience Institute, Morehouse School of Medicine Atlanta Georgia USA

**Keywords:** ASIC, cultured cells, photoreceptors, primate retina

## Abstract

Acid‐sensing ion channels (ASICs) are proton‐gated cation channels belonging to the epithelial Na + channel/degenerin superfamily. In the CNS, ASICs are involved in synaptic plasticity, learning/memory, and acidosis‐mediated injury. Previous studies showed that ASICs are expressed in rodent retina where activation likely participates in the phototransduction process and retinal integrity. However, there have been no studies examining the expression of ASICs in primate retina. Using molecular biology and patch‐clamp techniques, we explored the expression of ASICs in monkey retina and cultured monkey retinal cells, and the electrophysiological/pharmacological properties of ASICs in cultured amacrine/ganglion cells. RT‐PCR detected the expression of ASIC1a, 2a, 3, and 4 in intact monkey retina and cultured retinal cells. Patch‐clamp recordings showed transient ASIC currents with a pH 0.5 of 4.69. The currents were almost completely blocked by amiloride (100 μM) but were insensitive to PcTx‐1 (20 nM). The currents were potentiated by zinc (100 μM) and showed recovery from desensitization with a time constant of 0.18 s and were resistant to low conditioning pH with a pH 0.5 for steady‐state inactivation of 6.45. Our results for the first time demonstrate the expression of functional ASICs in primate amacrine/ganglion cells and suggest that ASIC currents in these cells are mediated predominantly by ASIC2a containing channels.

## 1 INTRODUCTION

ASICs belong to the epithelial Na^+^ channel/degenerin superfamily. They are activated by a decrease in extracellular pH and are present in both peripheral and central neurons (Krishtal, 2003; Waldmann et al., 1997; Wemmie et al., 2006). Four genes encode at least six ASIC subunits that form hetero and/or homo‐trimeric transmembrane channels permeable to Na^+^ alone or Na^+^ and Ca^2+^, depending upon subunit composition. In peripheral sensory neurons, activation of ASICs has been linked to nociception, mechanoreception, and taste transduction (Price et al., 2000; Sluka et al., 2003; Ugawa et al., 2002, 2003). In CNS neurons, ASICs are involved in synaptic plasticity, learning/memory, and fear conditioning (Du et al., 2014; Wemmie et al., 2002, 2003). In pathological conditions, activation of Ca^2+^ permeable ASIC1a channels has been shown to play an important role in neuronal cell death associated with various neurological disorders such as brain ischemia (Xiong et al., 2004), multiple sclerosis (Friese et al., 2007), and trauma (Yin et al., 2013). The expression and potential function of ASICs in retinal cells have also been explored. Previous studies in rodents showed that inhibiting ASIC1a channels by PcTx‐1, or gene knockdown, or deleting ASIC2a channels by gene knockout, altered the shape of the scotopic and photopic ERG a‐ and b‐waves (Ettaiche et al., 2004, 2006), suggesting that ASICs participate in the phototransduction process and in the early steps of signal transfer through the retina. In the present study, we used molecular biology to determine the expression of ASICs in the intact monkey retina and cultured retinal cells, and patch‐clamp recording to study ASIC currents in cultured monkey retinal amacrine/ganglion cells. We found that monkey retinal amacrine/ganglion cells express functional ASICs that are sensitive to amiloride but not PcTx‐1. The currents are potentiated by high concentrations of zinc and show rapid recovery from desensitization and low sensitivity to steady‐state inactivation. Our data suggest that ASIC currents in monkey retinal amacrine/ganglion cells are mediated predominantly by ASIC2a containing channels.

## 2 MATERIALS AND METHODS

### 2.1 Retinal isolation

Eyes from Rhesus macaques, ranging in age from 9 months to 22 years, were obtained from the Emory National Primate Research Center as part of their tissue distribution program. Collection and provision of tissues through the Emory National Primate Research Centers Biospecimen Distribution Program were approved by the Emory University Institutional Animal Care and Use Committee in accordance with the Animal Welfare Act and the U.S. Department of Health and Human Services “Guide for Care and Use of Laboratory Animals”. Eyes, collected from male and female primates, were sterilized by a 5 s immersion in 70% ethanol followed by 2 rinses with sterile balanced salt solution (BSS) containing (in mM): NaCl 139, KCl 3, HEPES 2, Na Pyruvate 1, MgCl_2_ 0.5, MgSO_4_ 0.5, NaH_2_PO_4_ 0.5, CaCl_2_ 1.8, NaHCO_3_ 1, Choline Cl 0.1, Phenol red 0.02, Glucose 16. The anterior portion of the eye globe was removed, and the vitreous extracted from the eye cup in BSS. The retinae were isolated from the eyecup, dissected into pieces measuring approximately 3–5 mm per side and maintained in BSS. Retinal pieces were used for RT‐PCR or generating dissociated cells as described in the following sections.

### 2.2 Reverse transcriptase‐polymerase chain reaction (RT‐PCR)

RT‐PCR was performed on intact retinal tissues and on cultured retinal cells as described in our previous studies (Li et al., 2010; O'Bryant et al., 2016). Total RNA was extracted with an RNeasy kit (Qiagen 74,106) according to manufacturer's instructions. For the cDNA synthesis, a 20 μL reaction volume containing 200 ng total RNA and appropriate amounts of RT buffer, dithiothreitol, dNTP, oligo(dT)_12–20_, RNase inhibitor, and reverse transcriptase (SuperScript II, Thermo Fisher, 18,064,014) was placed into a PCR tube. The mixture was incubated at 42°C for 50 min, followed by 15 min at 70°C to inactivate the reverse transcriptase. The oligonucleotide primers (Invitrogen) used are listed in Table 1. PCR reactions were carried out using 0.5 μL of cDNA as templates in PCR reaction buffer with 0.2 mM each dNTP, 0.5 U DNA polymerase (Taq DNA Polymerase, Thermo Fisher, 10,342,053), and 0.3 μM of each primer in a 10 μL reaction volume. The PCR amplification consisted of denaturation at 94°C for 3 min, then 35 cycles of denaturation at 94°C for 45 s, annealing at 60°C for 30 s, and extension at 72°C for 1 min. PCR products were separated via electrophoresis on a 1.5% agarose gel, and product bands were visualized with ethidium bromide/UV and later photographed.

**TABLE 1:** Primer sequences for different ASIC subunits.

Samples	Target	Forward primer sequences	Reverse primer sequences	Predicted size (bp)
Retinal tissues/cultures	ASIC1a	5′‐CTTCAAACCCAAACCCTTCA‐3′	5′‐TGGTCGATGAAAGGAGGTTC‐3′	342
	ASIC1b	5′‐ATGCTGGGACTGGATGAAAG‐3′	5′‐TGGTCGATGAAAGGAGGTTC‐3′	404
	ASIC2a	5′‐GCCTGCAACCTTCTAGCATC‐3′	5′‐ATGTGAGCCTCTGCTCCTGT‐3′	810
	ASIC2b	5′‐CCTCGAACCGCTTGCTCTAC‐3′	5′‐ATGTGAGCCTCTGCTCCTGT‐3′	693
	ASIC3	5′‐CGACTGCAGTTCAGCATCTC‐3′	5′‐AAGCAGCTCCGACATCTCAT‐3′	460
	ASIC4	5′‐ACAACCGCAACGAGACCTAC‐3′	5′‐CTCAGGCAGGGACTCTGTTC‐3′	316
	GAPDH	5′‐ATGCTGGCGCTGAGTACGTCGTG‐3′	5′‐TTACTCCTTGGAAGCCATGTGGG‐3′	743

### 2.3 Retinal cell dissociation

Isolated retinal cells were obtained by treatment of retinal pieces with papain following published methods (Lam, 1972; MacLeish et al., 1984). Briefly, retinal pieces were incubated at 37°C for 30–40 min, with gentle rocking, in a solution containing (in mM): NaCl 114, NaHCO_3_ 25, Na Pyruvate 1, KCl 3, NaH_2_PO_4_ 0.5, CaCl_2_ 0.5, Phenol red 0.02 combined with 7–10 units/mL of papain (Worthington; 3126), 2.7 mM of DL‐cysteine hydrochloride (Sigma, C‐9768), and 16 mM of glucose. The enzyme was removed by rinsing 3 times with BSS. Isolated cells were obtained via trituration of treated retina in BSS containing 32 mM glucose using a glass pipette. Cells were seeded onto coated glass coverslips in the wells of 35 mm plastic dishes (Mattek P35G‐0‐14‐C) and were cultured in serum‐free growth medium (see below) until used.

### 2.4 Dish preparation

Cells were grown on glass coverslips coated with antibody 9B5 (O'Malley & MacLeish, 1993). First, 35 mm glass bottom dishes (Mattek P35G‐0‐14‐C) were incubated with goat anti‐mouse affinity purified IgG (GαM, 0.1 mg/mL, Millipore, AP124‐K) in BSS, creating the first antibody layer. After incubation (>1 h), the dishes were rinsed with BSS to remove unbound antibody, then incubated (>1 h) at 4°C with monoclonal mouse antibody 9B5. The dishes were then rinsed (3×) with BSS, and the cells were seeded onto the coverslips at a density of approximately 1 × 10^6^ per dish. Cells settled undisturbed for 20 min, followed by the introduction of equilibrated culture medium. The presence of immobilized antibody 9B5 provided an adhesive substrate for the dissociated monkey retinal cells.

### 2.5 Cell culture

Retinal cells were maintained in a humidified 5% CO_2_ incubator at 37°C in serum‐free growth medium consisting of 1%–10% L‐15 (Sigma L‐5520) and 90%–99% of a salt solution containing (in mM): NaCl 114, HEPES 2, Na Pyruvate 1, MgCl_2_ 0.5, MgSO_4_ 0.5, NaH_2_PO_4_ 0.5, CaCl_2_ 1.8, KCl 3, NaHCO_3_ 25, Choline Cl 0.1, Phenol red 0.02, Glucose 16. The medium was supplemented with 10 μg/mL gentamicin (Gibco, 15,750–060) and 20 μg/mL bovine serum albumin (BSA, Sigma, A7030). Cells were fed twice a week by replacing 1 mL of the medium with fresh medium.

### 2.6 Electrophysiology

Electrophysiological recordings were performed in the whole‐cell mode of the patch‐clamp technique (Hamill et al., 1981) using Clampex 9.2 software (Axon), as described in our previous studies (Xiong et al., 2004). A holding potential of −70 mV was applied unless indicated otherwise. Cells were viewed using a Nikon TE2000 or Axiovert (Zeiss) phase contrast microscope and imaged with an Axiocam camera (Zeiss).

Cells were under constant perfusion with pH 7.4 extracellular fluid (ECF) containing (in mM): NaCl 140, KCl 5.4, CaCl_2_ 2, MgCl_2_ 1, Glucose 10, HEPES 20 (~320 Mosm/L, pH 7.4), at room temperature. A multibarrel fast perfusion system (SF‐77B, Warner Instruments) was used to achieve rapid changes of extracellular solutions.

Patch‐clamp pipettes were pulled on a Sutter P‐97 puller to a resistance of 3–5 MΩ. Internal solution (IS) contained (in mM): CsF 140, HEPES 10, EGTA 11, TEA 2, CaCl_2_ 1, MgCl_2_ 2, MgATP 4 (300 mOsm/L, pH 7.3). Free calcium was estimated to be ~20 nM.

### 2.7 Stock solutions of pharmacological reagents

PcTx‐1 (Psalmotoxin‐1; Peptide Institute Inc., Japan, 4435‐s), 20 μM in water; Amiloride (Sigma, A7410), 100 mM in DMSO; Zinc chloride (Fluka, 96,468), 100 mM in water. Stock solutions were diluted to the desired concentrations in ECF.

### 2.8 Data analysis

Data were expressed as mean ± standard deviation. Student's *t*‐test and ANOVA were used for statistical analysis where appropriate. A *p* value <0.05 is considered a statistically significant difference. The current responses were sorted, statistically analyzed, and graphed using Excel, Sigmaplot, and GraphPad software.

## 3 RESULTS

### 3.1 Transcriptional profile of ASICs in monkey intact retina and cultured retinal cells

**FIGURE 1:**
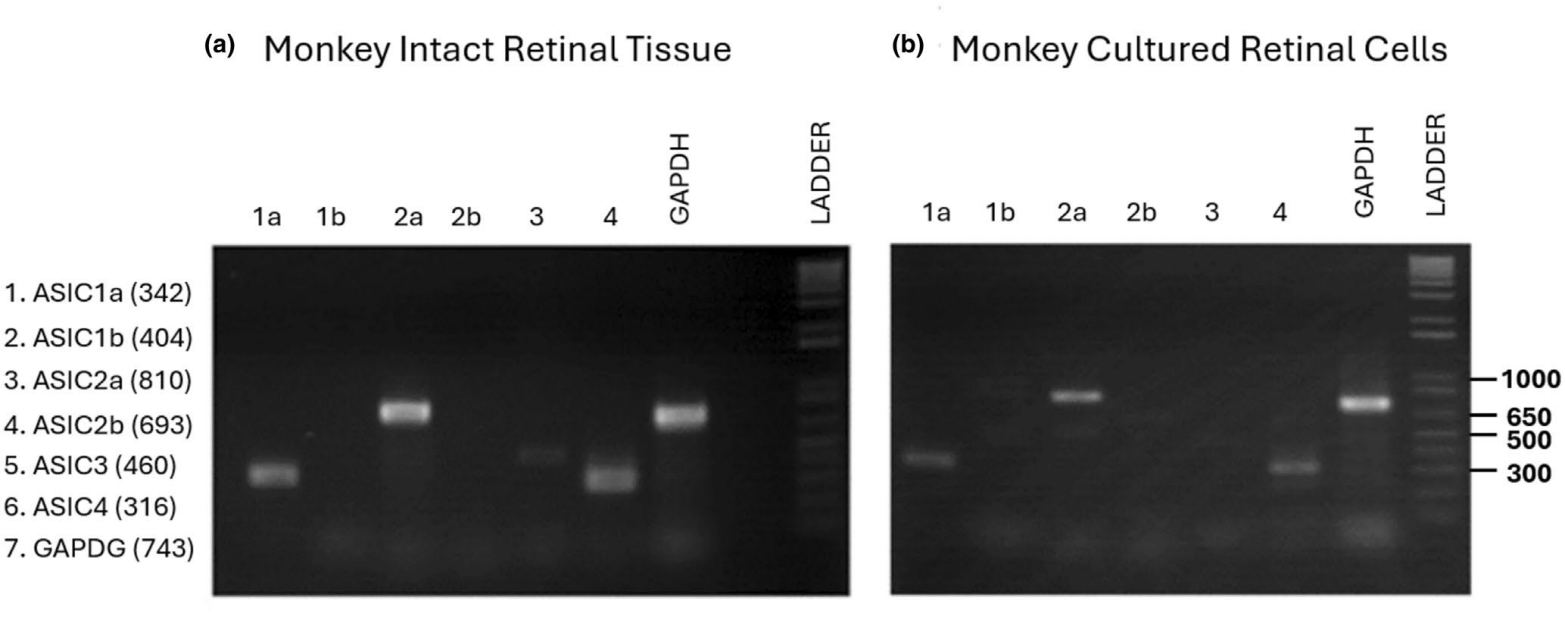
Expression profile of ASICs in primate retinal tissues and cultured retinal cells. (a) Expression of ASICs in intact primate retina. Intact retina was processed with primer pairs for ASIC1a, 1b, 2a, 2b, 3, and 4. Intact retinal tissue expressed ASIC1a, 2a, 3, and 4. (b) Expression of ASICs in cultured retinal cells. At 5 days in culture retinal cells expressed ASIC1a, 2a, and 4 but very weak ASIC3.

The ASIC subunit expression was first determined in intact monkey retina using RT‐PCR, as described in our previous studies (Li et al., 2010; O'Bryant et al., 2016). We used primer pairs to detect ASIC1a, 1b, 2a, 2b, 3, and 4 subunits. Figure 1a shows the expression of ASIC mRNAs in intact monkey retinal tissues where ASIC1a, 2a, and 4 were strongly expressed with weaker ASIC3 expression. To determine if our culture condition influenced expression profiles, we then examined the ASIC expression of retinal cells after 5 days in culture. Similar to the findings in the intact retina, ASIC1a, 2a, and 4 are clearly expressed in cultured retinal cells with very weak expression of ASIC3 (Figure 1b). The consistent expression profile in intact retina and cultured retinal cells suggests post‐dissociation survival of ASIC‐expressing cells under our culturing conditions.

### 3.2 Electrophysiological recordings of single amacrine/ganglion cells in culture

**FIGURE 2:**
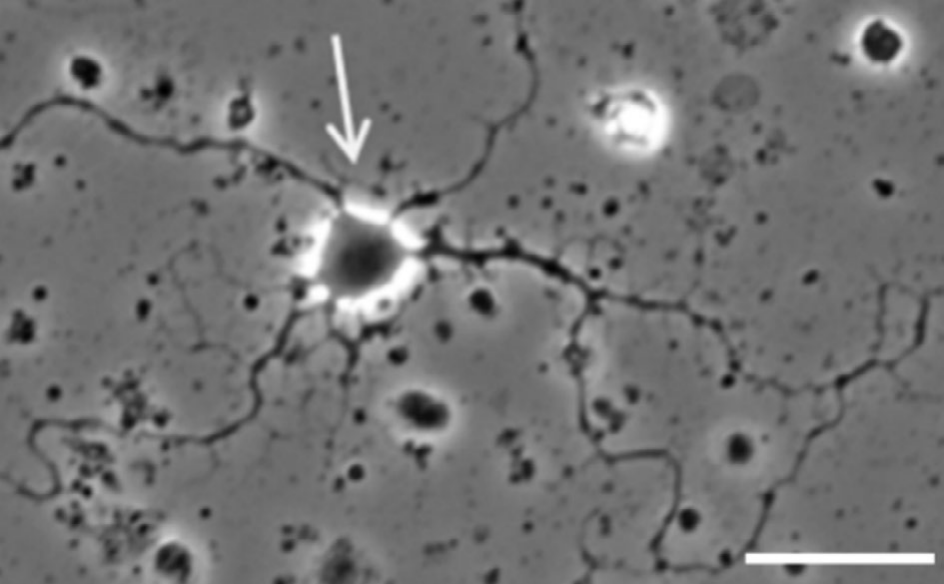
Phase contrast image of a living monkey amacrine/ganglion cell in culture. Scale bar: 25 μm.

After a few days in culture, monkey amacrine/ganglion neurons extended multiple processes from the cell soma (Figure 2). Since we had no definitive marker for either cell type, we classified these multipolar cells as amacrine/ganglion.

**FIGURE 3:**
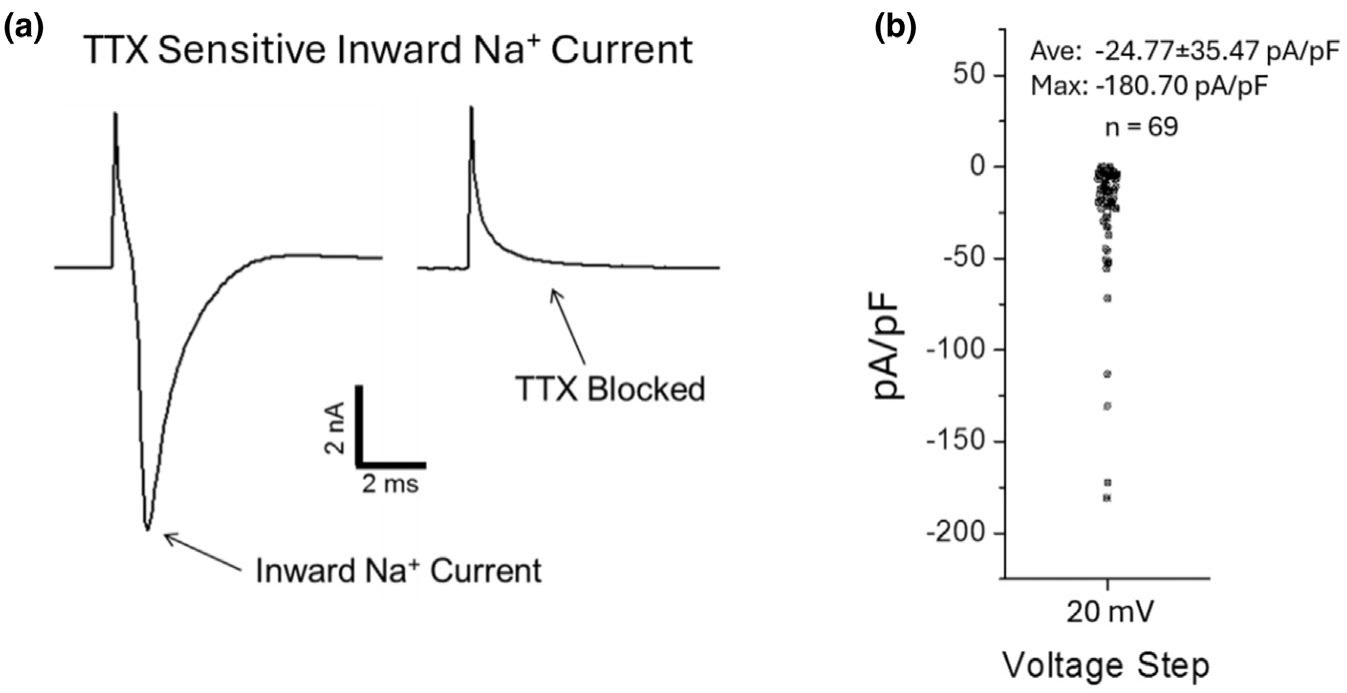
Fast TTX‐sensitive inward currents in monkey amacrine/ganglion cells. (a) An example of fast TTX (1 μM)‐sensitive inward current recorded at −20 mV from a holding potential of −70 mV (capacitance uncompensated). (b) Density of TTX‐sensitive inward current. The maximal density recorded was −180.7 pA/pF and the average current density in 69 cells was −24.77 ± 35.47 pA/pF.

We first established the excitability of these cells by recording fast, TTX‐sensitive inward currents activated by depolarizing pulses ranged between −70 and +60 mV from a holding potential of −70 mV. We found that 92 of 98 amacrine/ganglion cells showed detectable fast TTX‐sensitive inward currents when depolarized. The amplitude of the peak inward current varied considerably in different cells, with maximum peak current amplitude reaching as much as −8000 pA. Figure 3a shows an example of the fast inward current recorded at a depolarizing potential of −20 mV in an amacrine/ganglion cell which was completely and reversibly blocked by TTX (1 μM). Figure 3b shows a distribution of the density of TTX‐sensitive inward currents recorded in 69 amacrine/ganglion cells, with a maximum current density of −180.7 pA/pF and an average density of −24.77 ± 35.47 pA/pF. The variation of the current amplitude and dispersion of the current density values may be attributed to the many classes of amacrine/ganglion neurons recorded.

### 3.3 ASICs in monkey amacrine/ganglion cells

**FIGURE 4:**
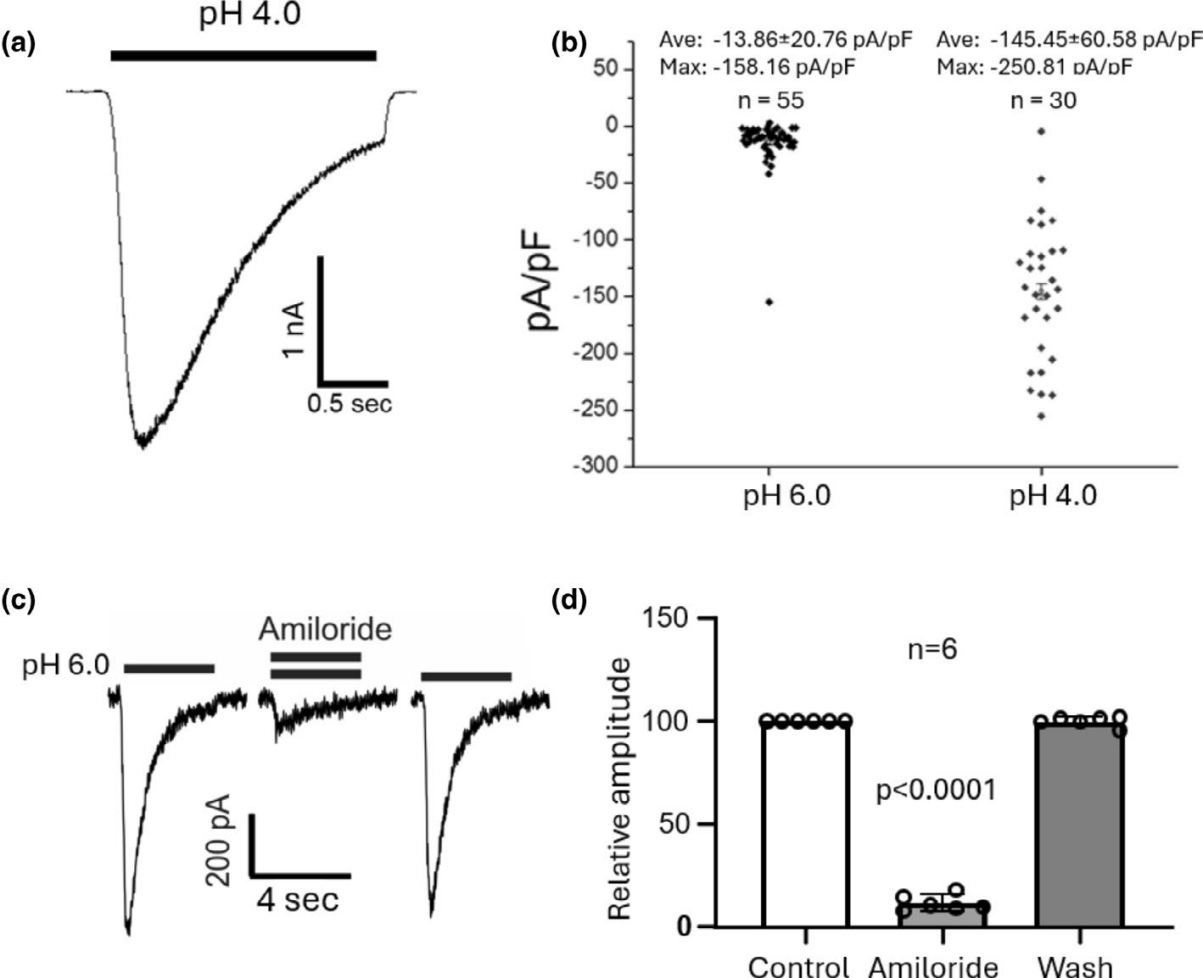
ASIC currents in monkey amacrine/ganglion cells. (a) Example trace showing ASIC current in response to pH drop from 7.4 to 4.0. (b) Density of peak ASIC currents activated by pH drops to 6.0 (*n* = 55), and 4.0 (*n* = 30). The average peak current density was −13.86 ± 20.76 pA/pF and −145.45 ± 60.58 pA/pF, respectively. (c) Example traces showing reversable blockade of ASIC currents by the non‐specific ASIC blocker Amiloride (100 μM). (d) Summary bar graph showing the effect of 100 μM amiloride on ASIC currents. Co‐application of amiloride reduced the peak amplitude of ASIC currents to 11.13 ± 5.09% of the control value (*n* = 6).

Next, we examined the ASIC currents in cultured monkey amacrine/ganglion cells. ASICs were initially activated by pH drops from 7.4 to 6.0 and 4.0. Of the 85 cells tested, 100% showed clear ASIC responses. Figure 4a shows an example of transient ASIC current activated at pH 4.0. Similar to the voltage‐gated TTX‐sensitive Na^+^ currents, the density of ASIC currents in monkey amacrine/ganglion cells varied considerably. The distributions of the peak current density activated by pH drops to pH 6.0 and pH 4.0 are shown in Figure 4b. The average peak current density activated at pH 6.0 and 4.0 was −13.86 ± 20.76 pA/pF (*n* = 55) and −145 ± 60.58 pA/pF (*n* = 30), respectively. As expected, ASIC currents in monkey amacrine/ganglion cells were reversibly blocked by 100 μM amiloride (Figure 4c,d).

### 3.4 Dose–response relationship of ASICs in monkey Amacrine/ganglion cells

**FIGURE 5:**
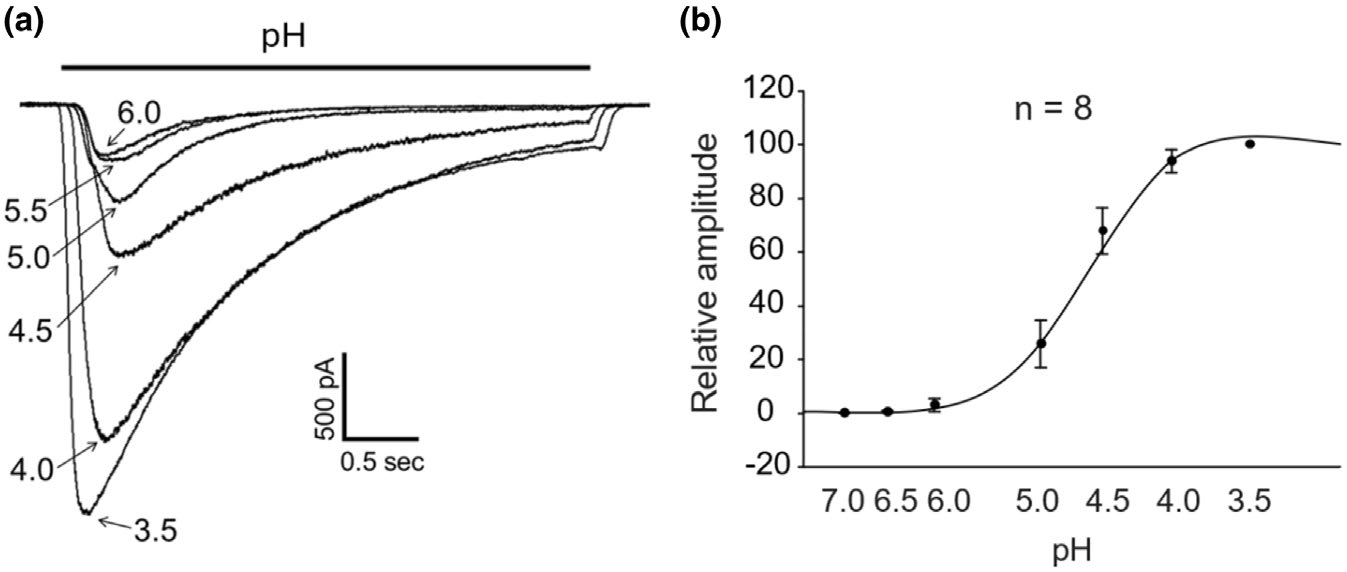
Dose–response relationship of ASICs in monkey amacrine/ganglion cells. (a) Example current traces showing dose‐dependent activation of ASICs in response to pH drops ranging from 6.0 to 3.5. (b) Summary graph showing dose–response relationship for ASICs (*n* = 8). The dose–response curve was fit to Hill equation with a pH_0.5_ of 4.69 ± 0.03.

Next, we constructed a dose–response relationship for ASICs in monkey amacrine/ganglion cells. As shown in Figure 5, the threshold pH for current activation is ~6.0, and the pH for maximal activation is ~3.5. Fitting of the dose–response curve with the Hill equation yielded a pH_0.5_ for activation of 4.69 ± 0.01 (*n* = 8).

### 3.5 Current–voltage relationship of ASICs in monkey amacrine/ganglion cells

**FIGURE 6:**
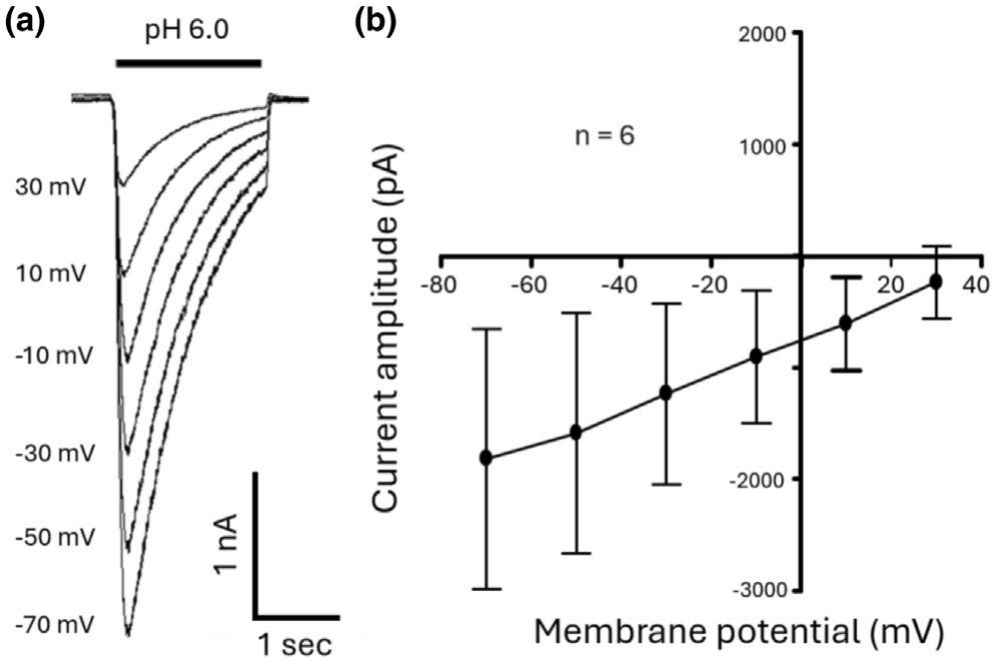
Current–voltage relationship of ASICs in monkey amacrine/ganglion cells. (a) Example ASIC currents activated by pH drops to 6.0 at holding potentials ranging from −70 mV to +30 mV. (b) Summary data showing the averaged current–voltage relationship (IV curve) for ASICs (*n* = 8).

We then constructed the current–voltage relationship for ASICs in cultured monkey amacrine/ganglion cells. Currents were activated by a pH drop from 7.4 to 6.0 at holding potentials ranging from −70 mV to +30 mV. Figure 6a shows example ASIC currents activated by pH drops from 7.4 to 6.0 at different holding potentials. Figure 6b shows the averaged ASIC current–voltage relationship of monkey amacrine/ganglion cells. As expected, the current–voltage relationship of ASICs in these cells was linear (*n* = 8).

### 3.6 Steady‐state inactivation and recovery from desensitization of ASICs in monkey amacrine/ganglion cells

**FIGURE 7:**
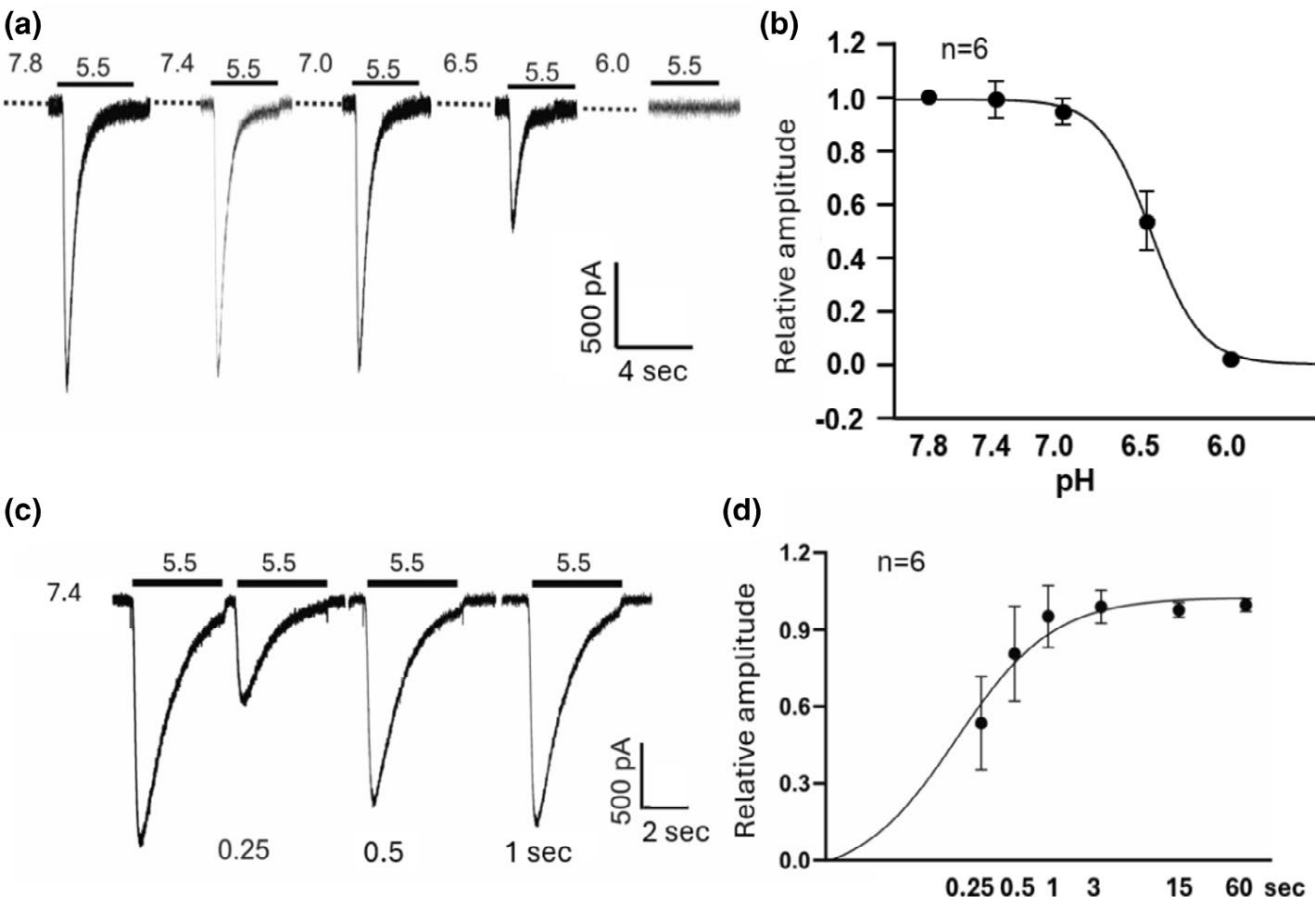
Steady‐state inactivation and recovery from desensitization of ASICs in monkey amacrine/ganglion cells. (a) Example current traces showing the steady state inactivation of ASICs. Cells were perfused with ECF at various conditioning pH (7.8, 7.4, 7.0, 6.5, and 6.0) for ~2 min before activating the ASICs by a pH drop to 5.5. (b) Summary graph showing the steady state inactivation curve of ASICs. The pH_0.5_ of the inactivation curve is 6.48 ± 0.09 (*n* = 5). (c) Example current traces showing time‐dependent recovery of ASICs from desensitization. The currents were activated by pairs of pH drops from 7.4 to 5.5 with different time intervals between the end of the first acid application and the beginning of the second acid application (0.25–60 s). (d) Summary data with an exponential fit showing the recovery rate of ASICs from desensitization (*n* = 6). The peak current amplitude activated by the second acid application is normalized to the amplitude of the first current in each pair and plotted against the time interval. The solid line is a single exponential fit to the data points. ↊ = 0.18 s.

Next, we examined the steady‐state inactivation of ASICs in monkey retinal amacrine/ganglion cells. Neurons were incubated in extracellular solutions at various conditioning pH values between 7.8 and 6.0 for ∼2 min before ASIC currents were activated by a drop in pH to 5.5. The amplitude of the currents activated with different conditioning pH values was normalized to the one activated with a conditioning pH of 7.8 (where there is no apparent inactivation) and plotted against the values of conditioning pHs. Unlike the finding in mouse cortical neurons where nearly 80% of the current was inhibited with a conditioning pH of 7.2 (Wang et al., 2006), about 50% of the current in monkey retinal amacrine/ganglion cells remains with a conditioning pH as low as 6.5 (Figure 7a,b). An average pH_0.5_ for steady‐state inactivation is 6.48 ± 0.10 (*n* = 6).

We then determined the recovery rate of ASICs from desensitization. ASIC currents were activated consecutively by pairs of low pH pulses (from 7.4 to 5.5) with various time intervals (0.25, 0.5, 1, 3, 15, and 60 s) between the end of the first and the beginning of the second acid exposure. The peak amplitude of the second current is then normalized to the first one and plotted against the time intervals between the two acid exposures. As shown in Figure 7c,d, ASIC currents in monkey retinal amacrine/ganglion cells recovered rapidly with a time constant of 0.18 ± 0.12 s (*n* = 6).

### 3.7 PcTx‐1 does not inhibit ASIC currents in monkey amacrine/ganglion cells

**FIGURE 8:**
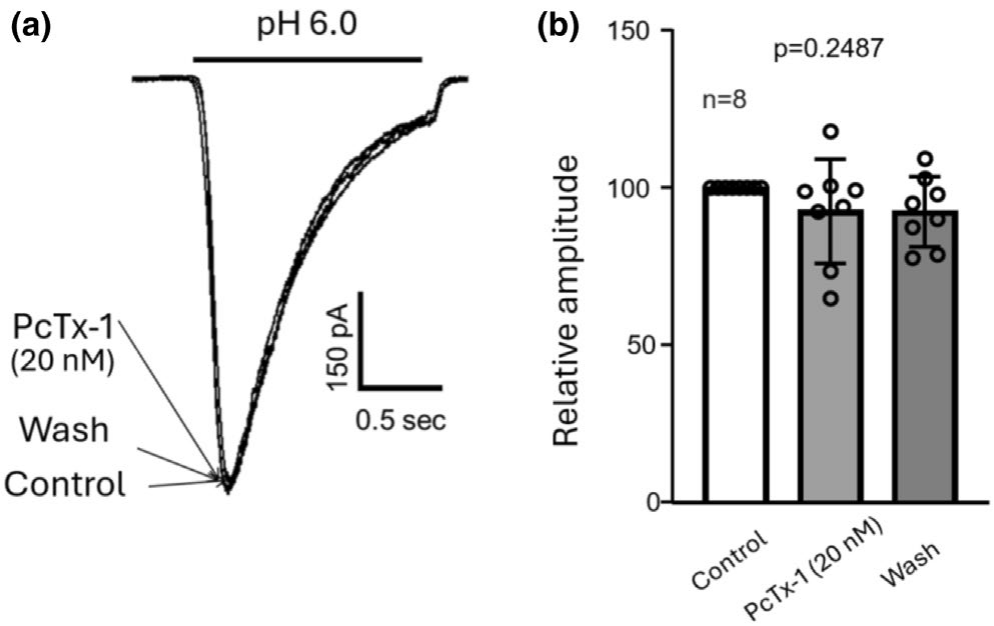
Lack of effect of PcTx‐1 on ASIC currents in monkey amacrine/ganglion cells. (a) Example current traces showing the lack of effect of PcTx‐1 on ASIC currents. (b) Summary bar graph showing the lack of significant inhibition on ASIC currents by PcTx‐1 (*n* = 9).

Next, we tested the effect of PcTx‐1, a specific inhibitor for homomeric ASIC1a and heteromeric ASIC1a/2b channels (Escoubas et al., 2000; Sherwood et al., 2011). The application of PcTx‐1 (20 nM) for up to 10 min did not significantly inhibit the ASIC currents in monkey amacrine/ganglion cells (Figure 8). In 9 amacrine/ganglion cells tested, the application of 20 nM PcTx‐1 for up to 10 min only slightly reduced the amplitude of ASIC currents to 92.98 ± 11.22 (*n* = 9, *p* = 0.2487). Additional tests performed in current‐clamp mode showed no effect of PcTx‐1 on acid‐mediated cell depolarization in amacrine/ganglion cells (*n* = 3, data not shown). The efficacy of the batch of PcTx‐1 was established by its blocking effects on ASIC currents in CHO cells transfected with ASIC1a subunits (data not shown). These results highlight the absence of homomeric ASIC1a or heteromeric ASIC1a/2b channels in cultured monkey amacrine/ganglion cells.

### 3.8 Zinc potentiation of ASIC current in monkey amacrine/ganglion cells

**FIGURE 9:**
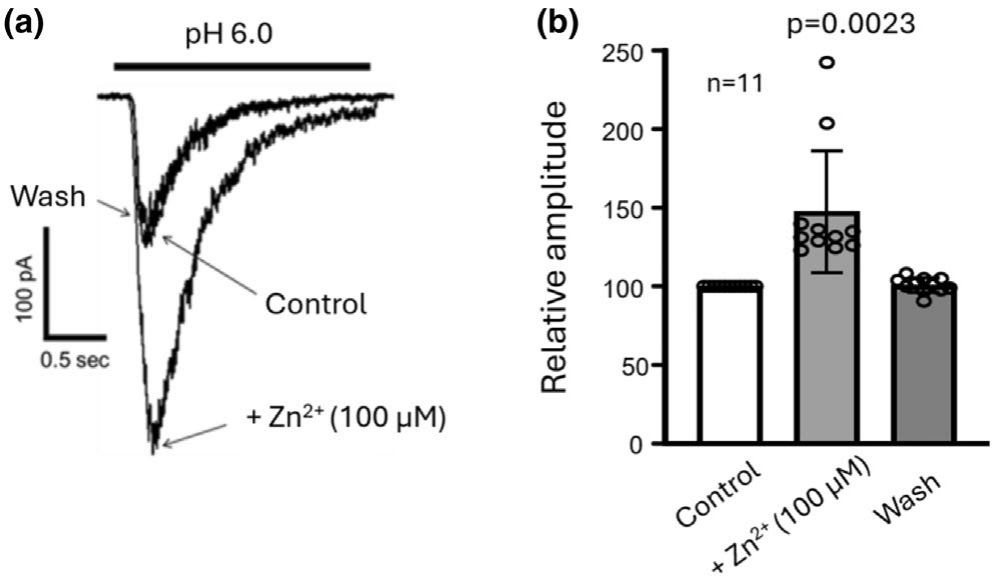
Effect of zinc on ASIC currents in monkey amacrine/ganglion cells. (a) Example current traces showing reversable potentiation of ASIC currents by 100 μM zinc coapplied with pH 6.0 solution (holding at −70 mV). (b) Summary data showing potentiation of ASIC currents by 100 μM zinc. The average zinc potentiation of ASIC currents is 47.00 ± 38.47% (*n* = 11).

To further determine the properties of the ASICs in monkey amacrine/ganglion cells, we examined the effect of zinc on the ASIC currents. Previous studies showed that zinc is an inhibitor of ASIC1a channels but a potentiator of the ASIC2a‐containing channels in mouse cortical and hippocampal neurons (Baron et al., 2001; Chu et al., 2004). In monkey amacrine/ganglion cells, zinc (100 μM) reversibly potentiated ASIC currents by 47.00 ± 38.47% (Figure 9, *n* = 11, *p* = 0.0023), suggesting the presence of ASIC2a‐containing channels in monkey amacrine/ganglion cells.

### 3.9 ASIC‐mediated depolarizations in monkey retinal amacrine/ganglion cells

**FIGURE 10:**
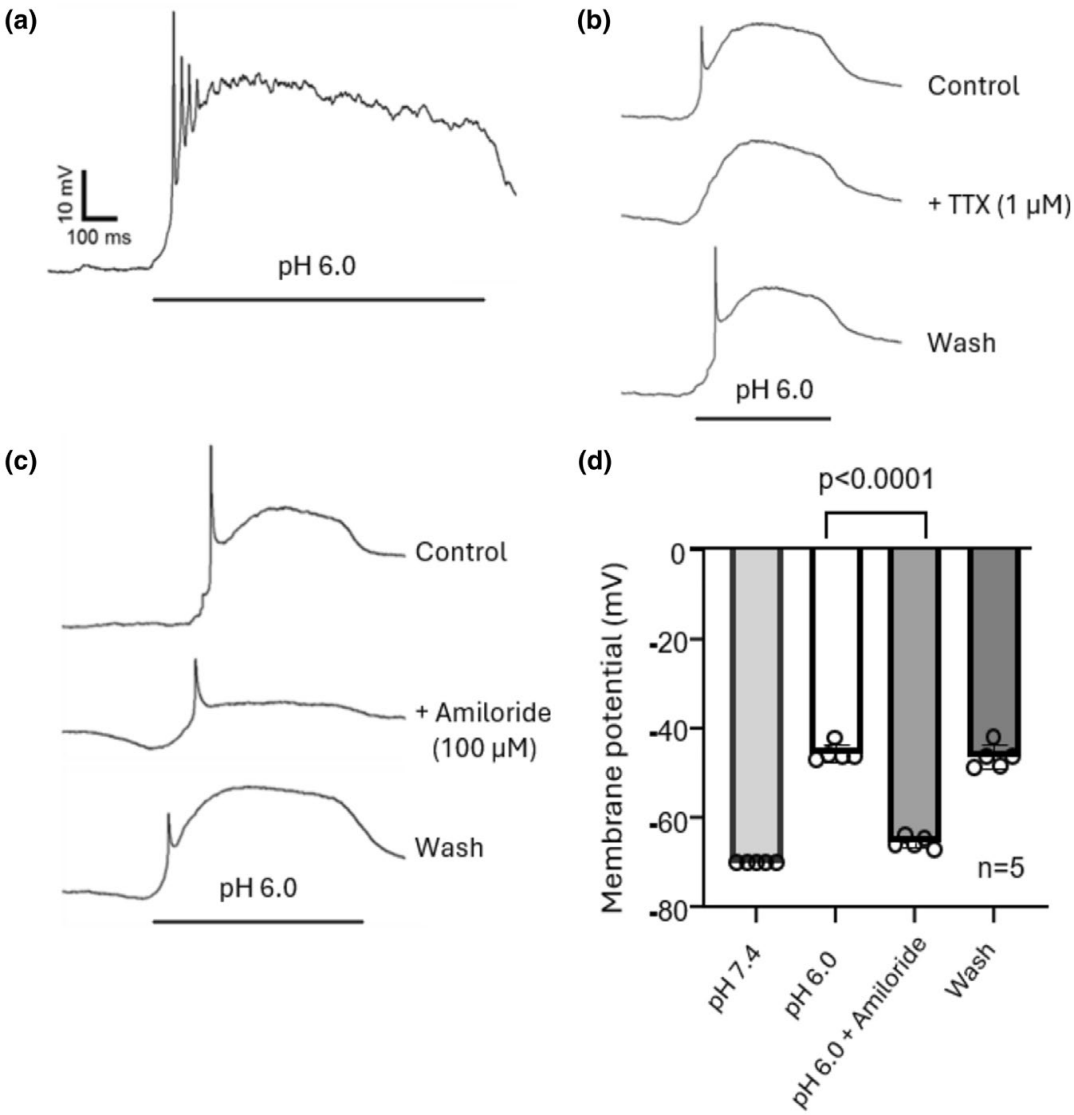
Activation of ASICs induces membrane depolarization and neuronal excitation in monkey amacrine/ganglion cells. (a) Activation of ASICs by dropping pH to 6.0 induced membrane depolarization accompanied by a train of action potential firing (resting membrane potential was −70 mV). (b) Acid‐induced firing of action potentials but not the slow depolarizations were abolished by TTX (1 μM). (c) The acid‐induced depolarization was reversibly abolished by amiloride (100 μM). (d) Summary data showing membrane depolarization induced by lowing pH to 6.0, which was inhibited by amiloride. *n* = 5.

Finally, we determined whether activation of ASICs in monkey retinal amacrine/ganglion cells can produce membrane depolarization or neuronal excitation. As shown in Figure 10a, activation of ASICs by dropping pH to 6.0 induced a large depolarization (>30 mV) which was accompanied by the firing of a train of action potentials. Application of TTX (1 μM) completely eliminated the action potential without affecting the slow membrane depolarization (Figure 10b). Application of amiloride (100 μM), however, reversibly suppressed the membrane depolarization (Figure 10c). These results indicate that activation of ASICs in monkey retinal amacrine/ganglion cells can produce membrane depolarization and neuronal excitation.

## 4 DISCUSSION

In the present study, we probed the expression profile of ASICs in intact primate retina and cultured retinal cells using molecular biology and studied the electrophysiological and pharmacological properties of ASICs in amacrine/ganglion cells using patch‐clamp techniques and morphological features for cell identification. We took advantage of our ability to maintain dissociated monkey retinal cells to investigate the presence of ASICs in these cells, which were taken from monkeys aged 2 yrs. to 22 yrs. and had thus completed their neuroanatomical development in situ prior to cell isolation. We did observe, however, that cells extended new processes during the period of experimentation. We have found that these cells retain natural sensitivity to neurotransmitters such as glutamic acid and GABA (not shown). The in vitro survival of primary neurons from adult primates is itself interesting since most, if not all, physiological studies using cultured cells are limited to early postnatal preparations. Our RT‐PCR and most of the electrophysiological/pharmacological characterization of ASICs focused on the cells between 5 and 10 days in culture. Whether the electrophysiological and pharmacological properties of ASICs in monkey retinal amacrine/ganglion cells changed with time in culture, as has been demonstrated in prenatal cultures of mouse cortical neurons (Li et al., 2010), was unclear. Also, the current study did not attempt to compare these properties of ASICs in cells cultured from the retina of monkeys of different ages.

This is the first report of the expression of ASICs in primate retina and in cultured retinal amacrine/ganglion cells, although the expression of ASICs in retina has been demonstrated in rats, mice, and rabbits (Brockway et al., 2002; Ettaiche et al., 2004, 2006; Lilley et al., 2004). Non‐human primates (NHPs) share vital similarities with humans, such as brain functional specialization that distinguishes them from rodents and makes them significantly better for translational investigations. Relevant to the current study, humans and NHPs are primarily visually oriented (Phillips et al., 2014), which makes the studies of ASICs in primate retinal cells more valuable.

We discovered using RT‐PCR that the intact primate retina has strong expression of ASIC1a, 2a, 4 and weak expression of ASIC3. Similarly, in cultured retinal cells we found that ASIC1a, 2a, and 4 are strongly expressed. This finding indicates that culturing conditions do not cause a major change in ASIC subunit expression. Our findings in intact monkey retina agree with earlier studies of retinal ASICs in rabbit (Brockway et al., 2002). Interestingly, in addition to ASIC1a, 2a, 3, and 4, Lilley et al. (2004) also detected expression of ASIC2b mRNA in rat retina.

We found that all amacrine/ganglion cells tested responded to low pH, in the range 6.0 to 3.5, with inward currents recorded at a holding potential of −70 mV. The low pH induced currents were reversibly blocked by 100 μM amiloride, a commonly used non‐specific inhibitor of ASICs, suggesting that the currents were mediated by ASICs. The pH dose response showed a maximal activation at pH 3.5 with a pH_0.5_ of 4.69. The finding of a low sensitivity of ASICs to H^+^ in monkey retinal amacrine/ganglion cells is similar to the result of Lilley et al (Lilley et al., 2004) in rat retinal ganglion cells (RGCs), which showed a pH_0.5_ for activation of 4.52 and that of Ettaiche et al. (2009) who reported a pH_0.5_ of 5.2 also in rat retinal ganglion cells. As anticipated for ASICs, the I‐V curve for acid‐activated currents in monkey retinal amacrine/ganglion cells showed a linear relationship, with a reversal potential close to E_Na_. This result indicates that Na^+^ was the main charge carrier for ASICs in monkey retinal amacrine/ganglion cells, a finding similar to that in rat retinal ganglion cells reported by Lilley et al. (2004). The time constant for recovery from desensitization of our monkey cells was 0.18 s compared with 1.55 s for mouse cortical neurons (Wang et al., 2006). This result suggests that the retina would be able to respond electrically to pH changes at a higher frequency than cortical neurons. In addition, the steady‐state inactivation of ASICs in monkey amacrine/ganglion cells had a pH_0.5_ of 6.48 compared with that of mouse cortical neurons that had a pH_0.5_ of 7.22. This finding suggests that the retinal cells might tolerate much lower pHs than mouse cortical neurons without inactivating ASICs.

To explore the subunit composition of the ASICs in monkey retinal amacrine/ganglion cells, we examined the ability of PcTx‐1 to inhibit the low pH induced currents. We observed that 20 nM PcTx‐1 had no detectable effect on ASIC currents. This result is consistent with the findings of Ettaiche et al (Ettaiche et al., 2006) that ASIC currents in rodent retinal ganglion cells are insensitive to PcTx‐1, suggesting the lack of homomeric ASIC1a and heteromeric ASIC1a/2b channels, which are known to be inhibited by PcTx‐1 (Escoubas et al., 2000; Sherwood et al., 2011).

Consistent with the presence of ASIC2a containing channels which are known to be potentiated by high concentrations of zinc (Baron et al., 2001), ASIC currents in all monkey retinal amacrine/ganglion cells were largely potentiated by 100 μM zinc. This result agrees with that of Ettaiche et al. (2006) who reported a large potentiation of ASIC currents by zinc in rat retinal ganglion cells but differed from that of Lilley et al (Lilley et al., 2004) who reported an inhibition of ASIC currents by zinc also in rat retinal ganglion cells. Based on the findings that the currents can be activated at ~pH 6.0, are insensitive to high concentrations of PcTx‐1 (20 nM), and are potentiated by high micromolar concentrations of zinc, we speculate that ASIC currents in monkey amacrine/ganglion cells are mediated primarily by heteromeric ASIC1a/2a channels. Of course, we cannot completely rule out the possibility that activation of homomeric ASIC2a channels might also contribute to the currents at very low pH values, for example, 5.0 or lower. Although our PCR result also showed the expression of ASIC3 which suggested the possible presence of homomeric ASIC3 and heteromeric ASIC1a/3 or ASIC2a/3 channels, the relatively low sensitivity of ASICs to pH drops in monkey retinal amacrine/ganglion cells (i.e., pH_0.5_ of 4.69, see Figure 5) argues against the contributions of homomeric ASIC3 and heteromeric ASIC3 containing channels, which have much higher pH_0.5_ values based on the studies of rodent ASICs (Verkest et al., 2022).

In addition to the studies on the electrophysiological/pharmacological properties and functions of rodent ASICs, a number of studies have described the properties and pharmacology of cloned human ASICs (Catarsi et al., 2001; Cristofori‐Armstrong et al., 2019; Delaunay et al., 2012; Verkest et al., 2022; Xu et al., 2018). These studies have shown clear differences in the properties of human ASICs as compared to those of rodent ASICs. For example, human ASIC1a was less inhibited by PcTX1 compared to rodent ASIC1a (Cristofori‐Armstrong et al., 2019), while human ASIC1a mediated stronger acid‐induced responses as compared to mouse ASIC1a (Xu et al., 2018). Moreover, heteromeric human ASIC2a/3 had a relatively lower pH_50_ for activation (e.g., 4.6) (Catarsi et al., 2001), as compared to that of rodent ASIC2a/3 (e.g., 6.1–5.6) (Verkest et al., 2022). Therefore, the exact subunit combination that shapes the ASIC currents in monkey retinal amacrine/ganglion cells remains to be determined.

We also performed experiments in current clamp to examine the effect of ASIC activation on membrane potential and neuronal excitation in monkey retinal amacrine/ganglion cells. We observed large depolarizations in response to low pH that were reversibly reduced by amiloride. The depolarizations frequently led to the generation of action potentials which were reversibly blocked by 1 μM TTX, indicating that they were generated by activation of voltage‐gated Na^+^ channels. Our finding that acid induces membrane depolarization and action potential firing in monkey amacrine/ganglion cells is similar to the result reported by Lilley et al in rat retinal ganglion cells (Lilley et al., 2004).

The role of ASICs in nervous tissue continues to be uncovered. In peripheral neurons, the physiological function of ASICs has been linked to nociception, mechanoreception, and taste transduction (Krishtal, 2003; Wemmie et al., 2006). Their role in the CNS, particularly in the retina, is less clear. However, studies have suggested that ASICs are involved in synaptic plasticity, learning/memory (Wemmie et al., 2002), and protons have been proposed as a neurotransmitter, at least in the amygdala (Du et al., 2014). Limited studies have also suggested their potential functions in the retina. Ettaiche et al. (Ettaiche et al., 2006), for example, showed that silencing ASIC1a alters cone‐mediated retinal function, while the knockout of ASIC2a increases light sensitivity, light sensitization, and retinal damage (Ettaiche et al., 2004). How the activation of ASIC2a‐containing channels contributes to light sensitivity, light sensitization, and retinal integrity is currently unclear, which warrants future investigations.

The most direct line of evidence for a role of ASIC in retinal function comes from the work of Ettaiche et al. (Ettaiche et al., 2006) which showed changes in the electroretinogram (ERG) waveform when ASIC1a was suppressed by antisense oligonucleotide injection or by the introduction of the ASIC1a blocker, PcTx‐1, into intact eyes. Injection of either antisense oligonucleotide or PcTx‐1 markedly reduced the amplitude of the photic ERG a and b waves. These results strongly suggest that ASIC1a channels are involved in cone function in the outer retina in a manner to be determined. The authors also reported a small but significant effect of the two agents on the a and b waves of the scotopic ERG, thereby pointing to a direct effect on rod function as well.

Under normal physiological conditions, light‐driven pH changes occur in the outer plexiform layer (OPL). In darkness, rod and cone photoreceptors release neurotransmitters at a maximal rate, causing acidification of the synaptic region of the OPL. Feedback from horizontal cells causes the release of GABA into cone synaptic terminals (Hirasawa & Kaneko, 2003) and alkalinization of that region occurs (Wang et al., 2014) upon horizontal cell activation. We speculate that the pH changes that occur are large enough to regulate ASIC1a channels that reside in the invaginating triad synaptic region based on findings in the amygdala (Du et al., 2014). We speculate that pH changes in the outer retina might spread to the inner retina where the amacrine/ganglion cells reside. What role the activation of ASICs in the inner retina plays is not clear, but the transient nature of the ASICs might contribute to the enhancement of light responses to flashes of light. Indeed, it has been shown that the release of transmitters from cat and rabbit retinae is increased significantly when flashing light is used to simulate the retina (Ehinger & Lindberg‐Bauer, 1976).

## ETHICS STATEMENT

This study was conducted in accordance with ethical guidelines governing general laboratory practice including transparency, full disclosure, and integrity. The ethical acquisition of monkey eyes was covered by the practices of the Emory National Primate Research Center.

## Data Availability

The data used in this study are available upon reasonable request from the corresponding author.
